# Stump Appendicitis

**DOI:** 10.5811/cpcem.2018.3.37730

**Published:** 2018-05-18

**Authors:** Alex C. Essenmacher, Emma Nash, Sarah K. Walker, Graeme J. Pitcher, Christopher T. Buresh, T. Shawn Sato

**Affiliations:** * University of Iowa Hospitals and Clinics, Department of Radiology, Iowa City, Iowa; † University of Iowa Hospitals and Clinics, Department of Emergency Medicine, Iowa City, Iowa; ‡ University of Iowa Hospitals and Clinics, Department of Surgery, Division of Pediatric Surgery, Iowa City, Iowa

## Abstract

Abdominal pain is a frequent problem encountered in the emergency department, and acute appendicitis is a well-recognized diagnosis. Laparoscopic appendectomy has become one of the most common surgical procedures in the United States. Patients with a history of appendectomy may experience recurrent right lower quadrant abdominal pain from an infrequently encountered complication that may occur when the residual appendix becomes obstructed and inflamed. We describe two cases of stump appendicitis in pediatric patients with a review of clinical and imaging findings and surgical management.

## INTRODUCTION

Abdominal pain is a common complaint in pediatric emergency visits, accounting for about 460,000 yearly visits for females under 15 years old and 314,000 for males under 15. Common entities such as appendicitis are diagnoses entertained early in the evaluation by emergency physicians; however, this diagnosis is often quickly ruled out in patients with a prior history of appendectomy. The provider must be familiar with complications of appendectomy such as retained stones and, as illustrated, stump appendicitis. Although rare, with a reported incidence of 1 in 50,000 appendectomies,^1^ under-recognition can cause a significant delay in diagnosis and treatment, leading to severe complications. We review two cases of children presenting with abdominal pain after previous laparoscopic appendectomies. These cases highlight the importance of being familiar with this unusual entity as well as the value of serial abdominal examinations and utilization of imaging including ultrasound.

## CASE REPORT

### Case One

An 11-year-old male presented to the emergency department (ED) with abdominal pain of one night duration causing difficulty with sleeping and ambulation. Of note, the patient denied loss of appetite, vomiting, and fever. Past surgical history was significant for appendectomy 19 months prior after presenting with similar symptoms and being diagnosed with appendicitis sonographically. There were no reported operative or postoperative complications.

Upon presentation the patient had not had a bowel movement in several days, and the initial leading differential diagnosis was constipation. Physical examination was significant for fever and localized peritonitis. Pertinent laboratory investigations at current presentation included leukocytosis of 13,300 per cubic millimeter (reference range 4,500–13,000), neutrophilia of 9,870 per cubic millimeter (reference range 1,700–7,500), and an elevated C-reactive peptide to 1.4 milligrams per deciliter (reference range <0.5). After antipyretics, repeat assessment showed a reduction in fever; however, the patient still had severe abdominal pain. A point-of-care ultrasound showed a normal-appearing gallbladder and no dilation of the common bile duct but demonstrated an aperistaltic mass in the right lower quadrant (RLQ).

**Image 1:**
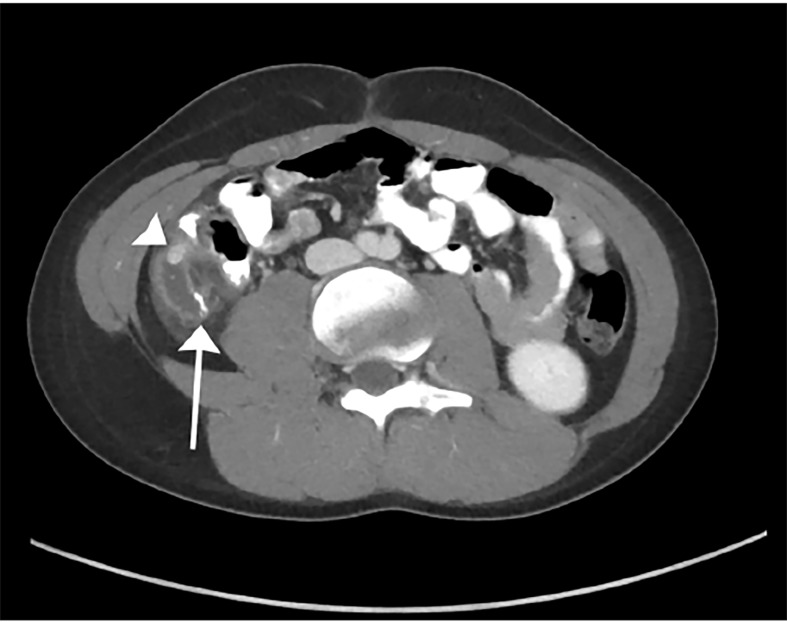
Contrast-enhanced computed tomography of the abdomen and pelvis, oblique axial plane, demonstrating surgical changes of appendectomy with staple lines at the blind end of the appendiceal stump (arrow). A high-density appendicolith (arrowhead) was obstructing the base of the appendiceal stump, which was surrounded by inflammatory changes.

After consulting with the pediatric surgery team, contrast-enhanced computed tomography of the abdomen and pelvis was performed and demonstrated surgical changes of appendectomy with staple lines at the blind end of the appendiceal stump. A high-density appendicolith was obstructing the base of the appendiceal stump, which was surrounded by mesenteric fat stranding (Image 1). Thickening of the appendiceal wall and the peritoneal reflection of the RLQ were additional findings consistent with acute appendicitis. There was no pneumoperitoneum. The patient was admitted and taken for laparoscopic surgery the next day. Surgical exploration revealed an inflamed appendiceal stump with pus in the right paracolic gutter. The appendiceal wall was very friable, and the stump required piecemeal removal during which time two appendicoliths were discovered in the lumen. The base was stapled flush with the cecum ensuring that no residual appendicoliths were present. The patient was discharged on postoperative day 3 and reported good recovery at follow-up appointments.

Pathology confirmed that the stump was necrotic, in two 2 cm long portions, with one portion containing a large appendicolith.

### Case Two

**Image 2:**
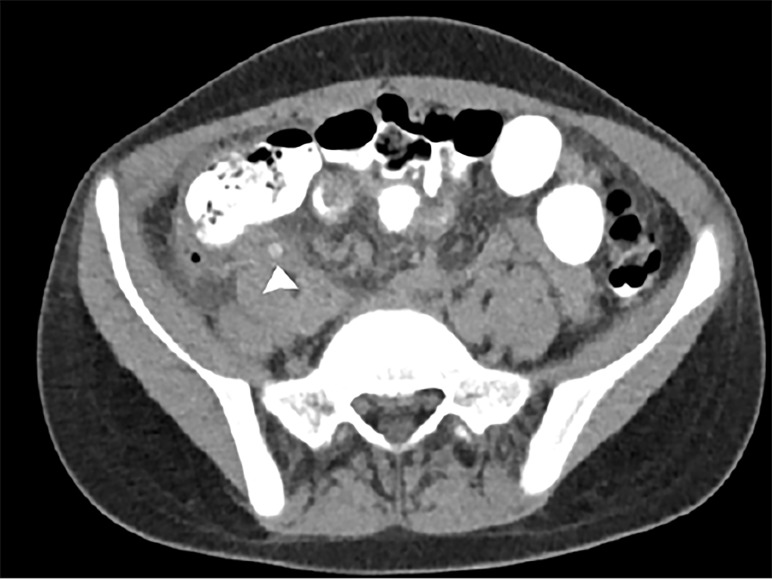
Axial contrast-enhanced computed tomography of the abdomen and pelvis in delayed phase demonstrating inflammation and extraluminal air in the right lower quadrant at the appendectomy site with high-density appendicoliths (arrowhead).

An 11-year-old female patient with a past medical history significant for appendicitis treated with laparoscopic appendectomy two months prior presented to a local ED with a one-day history of epigastric and right-sided abdominal pain, poor oral intake, and emesis. Prior to transfer to the university hospital, contrast-enhanced computed tomography of the abdomen and pelvis demonstrated a fluid collection in the right pericolic gutter at the site of surgical changes of appendectomy. The collection contained small stones (Image 2) and small foci of extraluminal air. There was also a small amount of frank pneumoperitoneum consistent with rupture of the appendiceal stump or dehiscence of the sutures.

Upon transfer, the patient was febrile and tachycardic. She was taken for laparoscopic appendectomy during which an inflamed, approximately 5 cm-long stump was encountered with an obvious appendicolith at its base adjacent to the cecum. The site of perforation was not readily evident, but there was evidence of recent peritoneal spillage and contamination. The previous staple line was readily apparent at the end of the stump. The appendectomy was completed by passing a stapling device proximal to the appendicolith and resecting the stump.

Pathology confirmed an inflamed, 5 cm appendix containing two large fecoliths. After gradual clinical improvement, she was discharged on postoperative day 4. Residual postoperative pain was well controlled with acetaminophen.

## DISCUSSION

Stump appendicitis is an uncommon entity; consequently it is rarely entertained as a diagnosis in a patient who has previously undergone an appendectomy, which may lead to a delay in diagnosis. One case series found perforation at the appendiceal stump in 60% of cases.^2^ Stump appendicitis may be significantly under-reported in the literature. Since 1945 there have only been about 60 cases reported in the English medical literature.^3^–^10^ It has been reported following laparoscopic as well as open appendectomy, and can occur many years after the original operation.^11^,^12^ It is thought to be more common following laparoscopic appendectomy,^2^ but a comparison to the open technique will become more difficult as that approach becomes less frequent. It is widely believed to be the result of a surgical illusion with respect to the actual location of the appendiceal base. This may be made more difficult by inflammatory changes and is probably more common after complicated appendicitis. Some authors suggest an appendiceal critical view^13^ similar to that described for cholecystectomy^14^ to avoid this problem.

**CPC-EM Capsule**

What do we already know about this clinical entity?

Stump appendicitis has an estimated incidence of 1 in 50,000 and can occur after both open and laparoscopic surgeries, months to years after initial removal.

What makes this presentation of disease reportable?

Stump appendicitis is not well described in the emergency medicine literature. These patients had an evolving abdominal exam consistent with appendicitis despite their surgical history.

What is the major learning point?

These cases highlight the importance of being familiar with this unusual entity, as well as the value of serial abdominal examinations and use of imaging including ultrasound.

How might this improve emergency medicine practice?

Increased awareness of this disease process among emergency physicians could prevent delayed diagnosis and complications.

The decision to use medical imaging in children can be difficult. While it is important to adequately rule out dangerous pathologies, it is also important to limit ionizing radiation doses in children. Ultrasound can be a screening tool to evaluate some etiologies of abdominal pain,^15^ but computed tomography with oral and intravenous contrast may be required for a definitive diagnosis in complicated and unusual cases such as these.

Pediatric patients presenting with abdominal pain often have an attributable, nonsurgical cause such as constipation or gastroenteritis. In the first case, the patient was initially afebrile, decreasing concern for a serious bacterial illness. However, he developed a fever throughout his ED course, and his abdominal exam became more concerning, illustrating the importance of observation and serial examination when the diagnosis is uncertain. Anchoring on a common diagnosis, such as constipation, and discharging the patient prior to the evolution of the fever could have devastating consequences.

## CONCLUSION

Stump appendicitis is an uncommon complication after appendectomy. It is important for physicians to be aware of this entity to ensure timely diagnosis and treatment of this unusual condition. With the increased utilization of laparoscopy for appendix resection, there may be an increased incidence of stump appendicitis after appendectomy, and it is important not to exclude appendicitis from the differential diagnosis based on prior history of appendectomy.

Documented patient informed consent and/or Institutional Review Board approval has been obtained and filed for publication of this case report.
